# Primary intracranial germ cell tumour originating from right brachium Pontis with hypertrophic Olivary degeneration: a case report

**DOI:** 10.1186/s12883-021-02238-0

**Published:** 2021-05-25

**Authors:** Yanong Li, Peng Wang, Jin Feng, Jiayi Wang, Jing Zhang, Xiaoguang Qiu

**Affiliations:** 1 Department of Radiation Oncology, Beijing Tiantan Hospital, Capital Medical University, 119, South 4th Ring West Road, Fengtai District, Beijing, China; 2 Department of Molecular Neuropathology, Beijing Neurosurgery Institute, Capital Medical University, Beijing, China

**Keywords:** Germinoma, Hypertrophic olivary degeneration, Brachium pontis, Chemoradiation therapy

## Abstract

**Background:**

Primary right brachium pontis germinoma with hypertrophic olivary degeneration (HOD) is extremely rare. A preoperative diagnosis is challenging due to the absence of characterized clinical and neuroimaging features, and biopsy should be considered.

**Case presentation:**

A 20-year-old male patient presented with a case of primary intracranial germinoma originating from right brachium pontis with HOD manifesting as ocular myoclonus, nystagmus in both eyes, ataxic gait and incoordination of the limbs. Magnetic resonance imaging (MRI) revealed an irregular patchy lesion with hyperintensity on T2-weighted images (T2WI) and T2 fluid-attenuated inversion recovery (FLAIR) without enhancement by gadolinium (Gd). Furthermore, a focal hyperintense nodule on T2WI in the left inferior olive nucleus (ION) of the medulla oblongata was considered hypertrophic olivary degeneration (HOD) based on the patient’s symptoms and neuroimaging findings. Due to suspected demyelinating disease and low-grade glioma (LGG), a biopsy was planned. The pathological diagnosis was germinoma. Subsequently, he received chemoradiation therapy, resulting in the improvement of neurological deficits and the disappearance of the lesion on MRI.

**Conclusion:**

A case of “Primary right brachium pontis germinoma with HOD” is reported for the first time. A preoperative diagnosis is challenging due to the fact of absence of clinical signs and symptoms and neuroimaging characteristics. However, patients can have favourable prognoses with appropriate evaluation and treatment.

**Supplementary Information:**

The online version contains supplementary material available at 10.1186/s12883-021-02238-0.

## Background

Central nervous system germ cell tumours (GCTs) represent a class of rare tumours with incidences ranging from 3.6% in North America to 15.3% in some areas of Asia, and they commonly occur in children and adolescents [1]. Although intracranial GCTs are located mainly in the pineal and suprasellar regions, 5 to 10% of GCTs are located in the basal ganglia. Moreover, other atypical sites include the cerebellar vermis [2], ventricular system [3], and medulla oblongata [4]. Here, we describe a highly rare case of a primary central nervous system germinoma originating from right brachium pontis with hypertrophic olivary degeneration (HOD). To our knowledge, this is the first presented case of primary central nervous system germinoma of brachium pontis.

## Case presentation

**Fig. 1:**
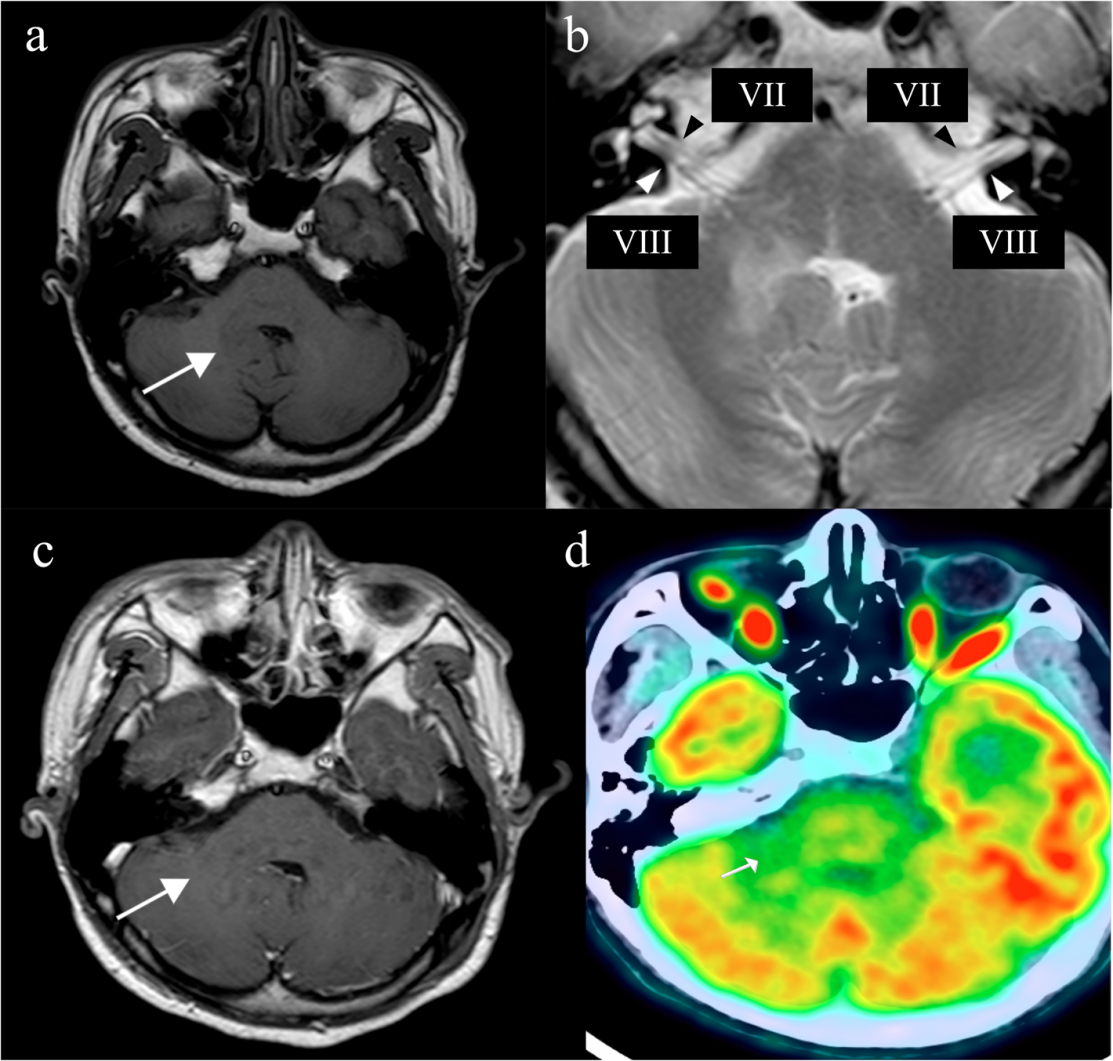
Axial T1 (**a**), T2 (**b**), and T1-weighted contrast-enhanced (**c**) MR images demonstrate an expansile ill-defined patchy lesion involving the right side of the middle cerebellar peduncle and dentate nucleus (*arrows*) as well as the displacement of the fourth ventricle. However, the ipsilateral facial nerve (*black arrowhead*) and vestibulocochlear nerve (*white arrowhead*) were not affected. The fused PET image (**d**) of the ^18^F-FDG PET/CT scan presents a decreasing metabolic lesion (SUVmax = 6.3) in the right brachium pontis that involves the ipsilateral cerebellar dentate nucleus (*arrows*)

**Fig. 2:**
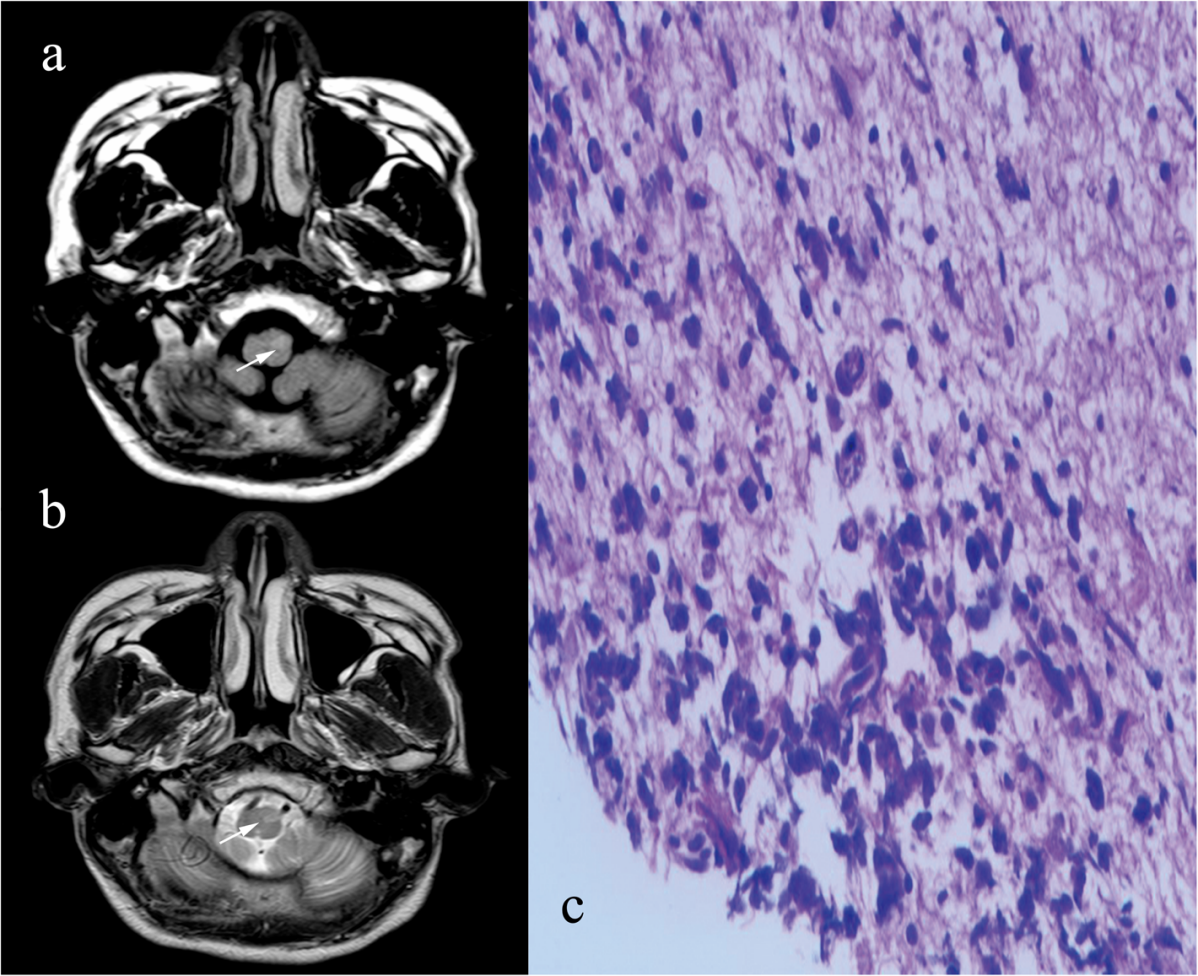
Axial T2WI (**a**) and FLAIR (**b**) MR images reveal subtle hyperintensity in the contralateral inferior olive nucleus (arrows). Histopathological analysis (**c**) reveals a cluster of pleomorphic cells with oval nuclei (immunohistochemistry, × 100)

**Fig. 3:**
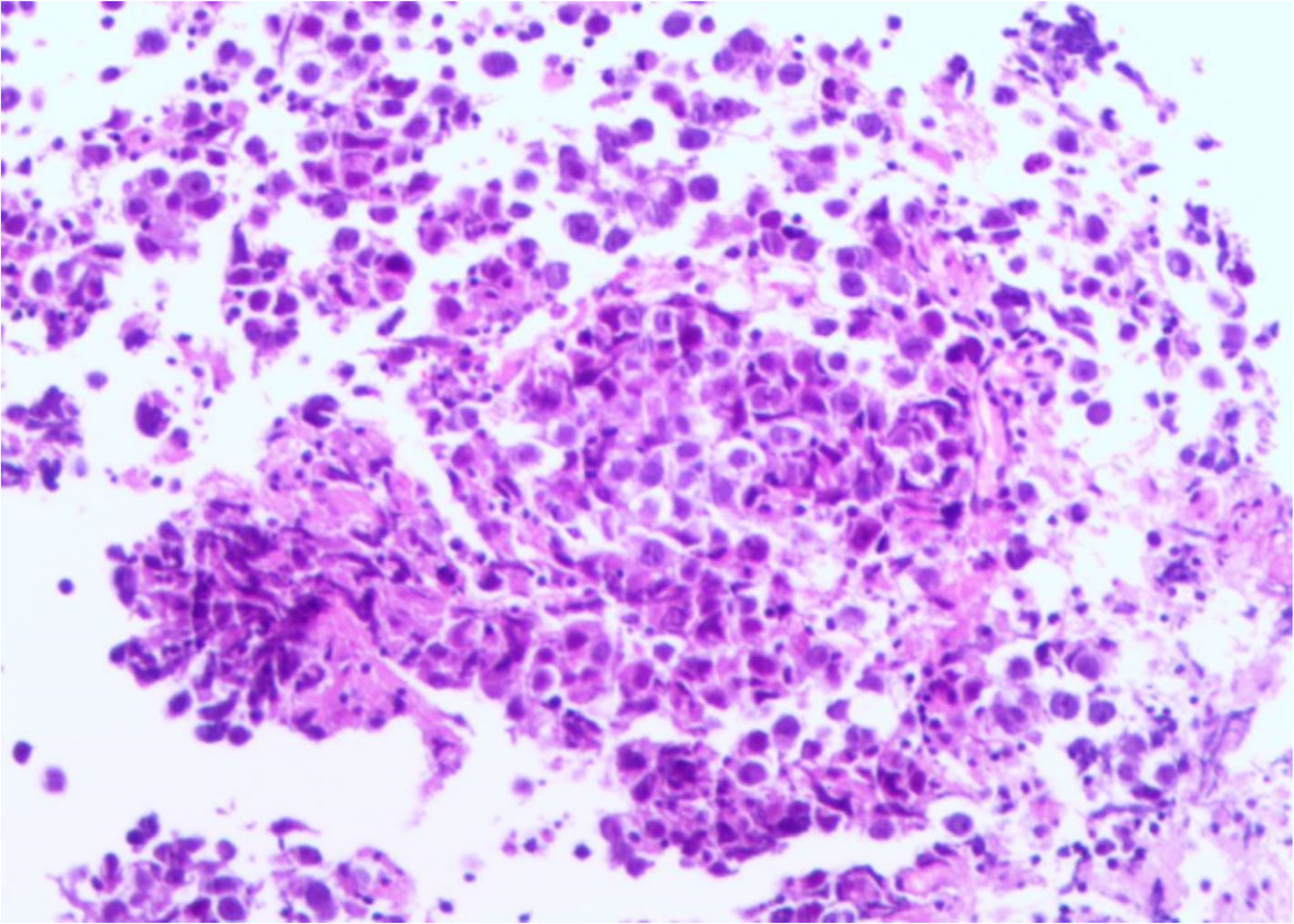
Histology of the surgical specimen, indicating large cells with is that regression merely reflected shrinkage with a polygonal or spheroidal nuclei and minimal lymphocytic infiltration (Hematoxylin & eosin stain, X400)

A 20-year-old male who had a resent history of 10-month history of dizziness after strenuous activities, headache and vomiting and a 5-month history of progressive deterioration of fine movement of the right extremitiy. A neurological examination was performed; the patient’s presented ocular myoclonus, nystagmus in both eyes, and ataxic gait, positive the finger-to-nose and heel-to-shin tests. Initial brain MRI, reviewed an irregular patchy 1.6-cm isointense to hypointense lesion on T1-weighted image (T1WI) (Fig. 1a), no enhancement after contrast (Fig. 1c), and a hyperintense lesion on T2-weighted image (T2WI) (Fig. 1b) in the right brachium pontis involving ipsilateral cerebellar dentate nucleus was detected. The fourth ventricle appeared asymmetric on axial view. Additionally, a focal 0.7-cm hyperintense nodule on T2WI in the left inferior olive nucleus (ION) of the medulla oblongata was considered hypertrophic olivary degeneration (HOD) according to the patient’s symptoms and neuroimaging findings (Fig. 2a and b). With suspected demyelinating disease and low-grade glioma (LGG), a biopsy was arranged. Operatively, the lesion in the right brachium pontis was solid and grey-coloured, and the biopsy result was GCT. In contrast to the initial suspicious diagnosis, the pathological diagnosis of the main lesion in the right brachium pontis was germinoma. Focal expression of PLAP, OCT3/4, and c-Kit was acquired, and alpha- fetoprotein (AFP), CD30 and placental alkaline phosphatase were not significant (Fig. 2c: immunohistochemistry, IHC; & Fig. 3: Hematoxylin & eosin stain, H&E). Serum AFP (2.99 ng/mL) and β-hCG (0.31 mU/mL) levels were within the normal range. The brain ^18^F-fluorodeoxyglucose positron emission tomography/computed tomography (^18^F-FDG PET/CT) examination found a hypodense lesion in the right brachium pontis showing slightly decreased metabolism with a maximum standardized uptake value (SUVmax) of 6.3 (Fig. 1d). After establishing the pathological diagnosis, the patient first received platinum-based chemotherapy followed by radiation therapy, including 23.4 Gy to the whole brain and 12.6 Gy to the tumour (total, 36 Gy), and then completed the remaining 4 cycles of chemotherapy based on the same regimen. Six months after chemoradiotherapy, the follow-up MRI (Fig. S1) showed no specific tumour residue and no evidence of dissemination, and the hyperintense nodule on T2W image in the left ION of the medulla oblongata had resolved. The patient is currently undergoing rehabilitation training, and the latest evaluation of muscle strength testing showed that the power of the right shoulder abductors and the elbow flexors was 4, and the power of the elbow extensors and wrist extensors was 4+. The power of hip flexors, knee extensors, dorsiflexion, great toe extensor, and plantar flexors was 4-. The last follow-up was 34 months after the end of treatment, and MRI revealed that the HOD had disappeared (Fig. S2). The patient was in good condition with no signs of recurrence.

## Discussion and conclusion

Germinoma, primarily arising in brachium pontis with HOD, is an enigma. Based on the brain MRI scan, the tumour involved right brachium pontis with abnormal appearance of a hypertrophic contralateral inferior olivary nucleus (ION), which may occur secondary to pontine haemorrhage, tumour, demyelination, infection, or postsurgery. In the present case, the lesion involved the dentate nucleus, the dentato-rubro-olivary component, and the main pathway of the triangle of Guilain and Mollaret, which constituted the connection between the red nucleus, ipsilateral inferior olivary nucleus, and contralateral dentate nucleus. The pathological manifestations of HOD include vacuolation and enlargement of neurons, astrocytic hyperplasia, proliferation with gliosis and demyelination [5], and hyperintensity of the olive on T2WI can appear as early as 3 to 4 weeks [6]. Only a few studies have reported on MR imaging and ^18^FDG-PET findings of basal ganglia GCTs. However, intracranial germ cell tumours originating from the brachium pontis are the same as basal ganglia GCTs, and they all belong to atypical site GCTs. Moon WK et al. described several different MRI manifestations of germ cell tumours arising from the basal ganglia. These manifestations range from subtle nonenhancing patchy lesions to enhanced large masses [7]. On ^18^FDG-PET images, Vali R et al. showed that the metabolism of the tumour was reduced, and the activity of the ipsilateral cerebral cortex was also decreased compared to that of the contralateral cerebral cortex [8]. Chih-Hsiang B. Yu reported a case of a fourth ventricle germ cell tumour and described the ^18^FDG-PET manifestation. The imaging results showed that the metabolic activity of ^18^F-FDG was significantly increased [9]. There is no evidence why germ cell tumours with the same pathology have different ^18^FDG-PET manifestations, and whether this difference is related to different sites, increased tumour markers and immunohistochemical types requires further study. Furthermore, because many of the nerve fibres from the dentate nucleus to inferior olive are primarily inhibitory GABArgic, damaging the dentato rural pathway results in loss of inhibitory control with consequent hyperactivity of the olivary neurons leading to abnormal involuntary movements, such as the present patient’s palatal myoclonus, choreodystonia, ataxia, and dysarthria [6].

Germinomas are sensitive to radiation therapy, and patients with newly diagnosed intracranial GCTs should be considered for radiation therapy and synergism with chemotherapy to decrease the dose and volume of irradiation [10]. Whole-brain irradiation (WBI) or whole-ventricular irradiation (WVI) fields with a boost are administered at a dose from 24 to 30 Gy, and cerebrospinal irradiation (CSI) ranged from 30 to 36 Gy with a boost to 45 to 50 Gy [11].

Primary right brachium pontis germinoma with HOD is reported for the first time. A preoperative diagnosis is challenging on account of the absence of representative clinical and neuroimaging characteristics. In contrast, the patient could have favourable prognoses with appropriate evaluation and therapy.

## Supplementary Information

**Additional file 1: Figure S1.** The follow-up MRI (16 months later). Axial T2WI (A) and FLAIR (B) MRI demonstrate that the lesion in the right brachium pontis disappeared. Axial T2WI (C) and FLAIR (D) demonstrate that the hypertrophic left ION disappeared.

**Additional file 2: Figure S2.** Axial T1WI (a) and T2WI (b) MR images revealed HOD after chemoradiotherapy (red arrow).

## Data Availability

Not applicable.

## Abbreviations

HOD: Hypertrophic olivary degeneration
MRI: Magnetic resonance imaging
T2WI: T2-weighted images
FLAIR: Fluid-attenuated-inversion-recovery
Gd: Gadolinium
ION: Inferior olive nucleus
LGG: Low-grade glioma
GCTs: Germ cell tumours
T1WI: T1 weighted image
AFP: Alpha-fetoprotein
IHC: Immunohistochemistry
^18^F-FDG PET/CT: ^18^F-fluorodeoxyglucose positron emission tomography/computed tomography
SUVmax: Maximum standardized uptake value
WBI: Whole-brain irradiation
WVI: Whole-ventricular irradiation
CSI: Cerebrospinal irradiation
