# Supplementary figures and images for: Primary intracranial germ cell tumour originating from right brachium Pontis with hypertrophic Olivary degeneration: a case report
Source: BMC Neurol. 2021 May 25;21:210. doi: 10.1186/s12883-021-02238-0 (PMC8146987; doi:10.1186/s12883-021-02238-0)

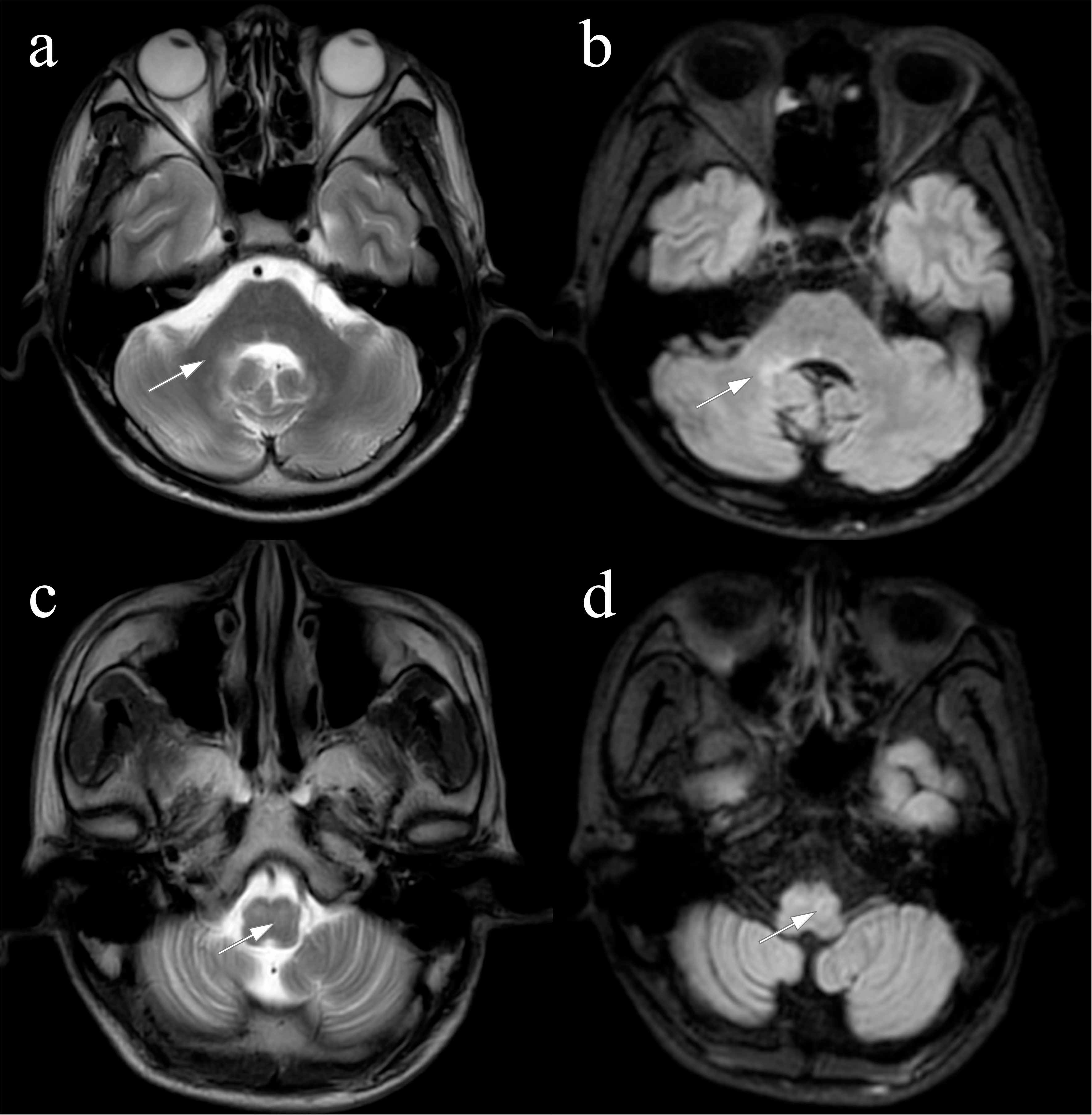

Supplement: Supplementary file 1 — Additional file 1: Figure S1. The follow-up MRI (16 months later). Axial T2WI (A) and FLAIR (B) MRI demonstrate that the lesion in the right brachium pontis disappeared. Axial T2WI (C) and FLAIR (D) demonstrate that the hypertrophic left ION disappeared. [file 12883_2021_2238_MOESM1_ESM.tiff]

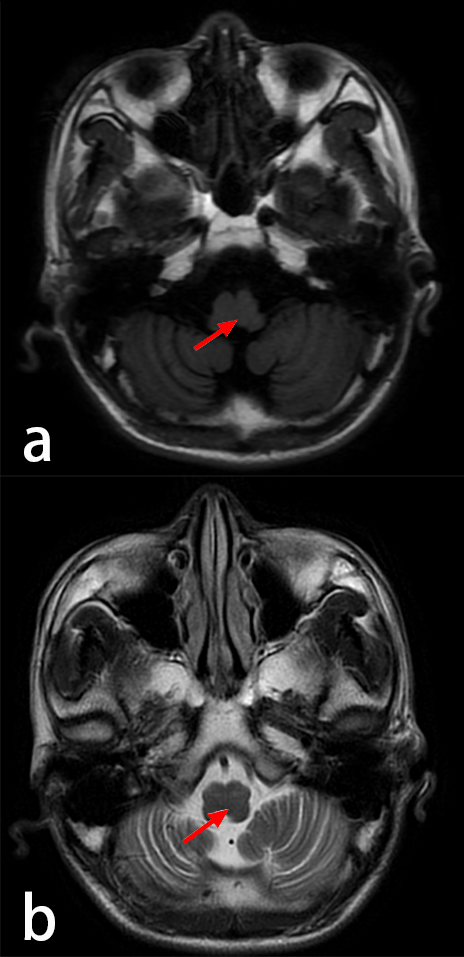

Supplement: Supplementary file 2 — Additional file 2: Figure S2. Axial T1WI (a) and T2WI (b) MR images revealed HOD after chemoradiotherapy (red arrow). [file 12883_2021_2238_MOESM2_ESM.tif]
